# Benign Mixed Tumour of Eccrine Origin Presenting as Upper Lip Swelling: A Report of a Rare Case and Literature Review

**DOI:** 10.7759/cureus.83865

**Published:** 2025-05-10

**Authors:** Hari Krishnan B, Prasanna Kumar, Karthikeyan Selvaraj, Sasikumar Pattabi

**Affiliations:** 1 General Surgery, Sree Balaji Medical College And Hospital, Chennai, IND; 2 General Surgery, Sree Balaji Medical College and Hospital, Chennai, IND

**Keywords:** chondroid syringoma, eccrine tumor, mixed adnexal tumor, myoepithelial cells, upper lip swelling

## Abstract

Benign mixed tumors of eccrine origin are rare cutaneous neoplasms characterized by both epithelial and mesenchymal differentiation. These tumors, arising from eccrine sweat glands, often present as slow-growing, solitary nodules and are commonly misdiagnosed due to their nonspecific clinical appearance. We report the case of a 47-year-old male patient with a seven-month history of a firm, ill-defined upper lip swelling initially suspected to be a sebaceous cyst. Surgical excision followed by histopathological evaluation revealed features consistent with a benign mixed tumor of eccrine origin, showing prominent myoepithelial components, ductal structures, and chondromyxoid stroma. Mixed adnexal tumors of eccrine origin, including eccrine spiradenomas and chondroid syringomas, are rare but important differentials for mucocutaneous swellings. Histopathology remains the gold standard for diagnosis. Surgical excision offers an excellent prognosis, with low recurrence rates. This case underscores the importance of considering rare adnexal tumors in the differential diagnosis of lip swellings. Early recognition, complete surgical excision, and histological assessment are pivotal in achieving favorable outcomes.

## Introduction

Benign adnexal tumors of eccrine origin are a subset of skin neoplasms derived from eccrine sweat glands, which play a crucial role in thermoregulation and are widely distributed across the body [1]. These tumors are typically well-differentiated and may exhibit a mixture of epithelial and mesenchymal features, leading to their classification as "mixed tumors." Although histologically benign, their diverse presentations and morphological overlap with other dermal lesions can make diagnosis challenging.

Common benign eccrine tumors include eccrine spiradenoma, which typically presents as a painful, solitary dermal nodule, often on the trunk or extremities. Chondroid syringoma (also known as cutaneous pleomorphic adenoma), a rare tumor resembling its salivary gland counterpart, is characterized by the presence of glandular epithelial structures within a chondromyxoid or cartilaginous stroma. Syringoma usually presents as multiple, small, firm papules, commonly around the periorbital region [2].

Due to their rarity and non-specific clinical appearance, especially when arising in unusual locations such as the lip, these tumors are often misdiagnosed preoperatively, frequently mistaken for epidermoid cysts, mucoceles, or other adnexal lesions. Definitive diagnosis relies on histopathological examination, which reveals their biphasic architecture.

In this report, we present a rare case of chondroid syringoma of the lip, supplemented by a review of existing literature, to enhance recognition and understanding of this uncommon adnexal tumor [3].

## Case presentation

A 47-year-old male patient presented with a gradually enlarging, painless swelling over the upper lip that had been progressively increasing in size over the past seven months. The swelling was not associated with any systemic symptoms such as fever, weight loss, or discharge. Clinical examination revealed a firm, ill-defined swelling approximately 2 × 2 cm in size, located in the mucosal region of the upper lip. The surface was smooth, and the overlying skin appeared stretched and non-pinchable, with no signs of ulceration or inflammation. The lesion was mobile over underlying structures but relatively fixed to the overlying mucosa.

High-resolution ultrasonography of the upper lip demonstrated a well-defined cystic lesion measuring approximately 1.5 × 0.7 cm, located in the cutaneous plane. The lesion had internal echoes suggestive of debris, and vascularity was normal with preservation of the muscular plane. The imaging features were initially suggestive of a sebaceous cyst.

**Figure 1:**
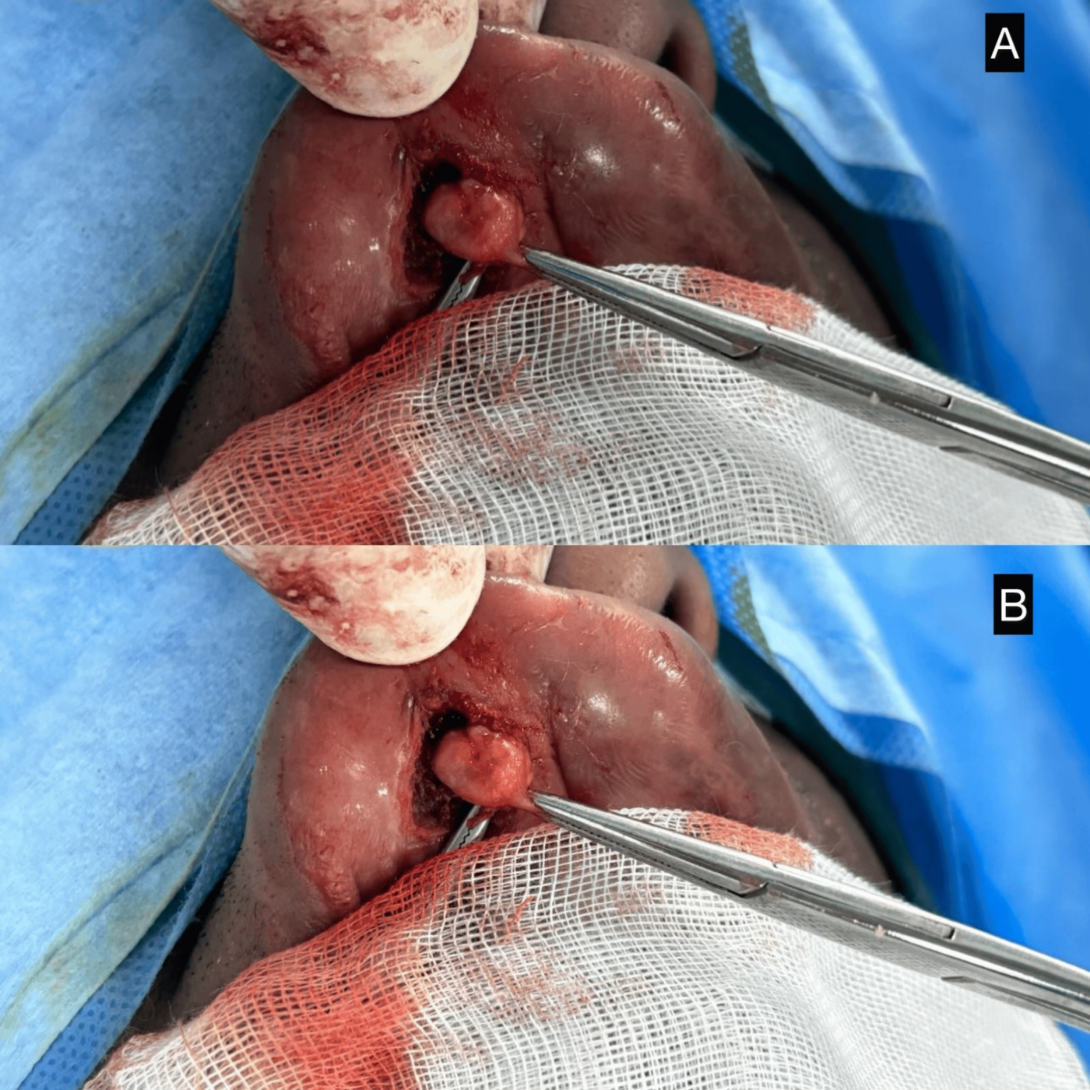
Intraoperative clinical images demonstrating the excision of a firm nodular lesion from the upper labial mucosa A and B: The lesion is seen being delivered and dissected from the sinus tract using forceps. The nodular mass appears well-defined, consistent with a benign adnexal tumor.

Given the persistence and appearance of the lesion, the patient underwent surgical excision under local anesthesia. An intraoral approach was utilized to minimize cosmetic scarring. The lesion was excised in toto, and the surgical site was closed in layers using 3-0 monocryl sutures (Figures 1A, 1B). Gross examination of the specimen revealed a well-circumscribed, firm lesion with a smooth external surface.

**Figure 2:**
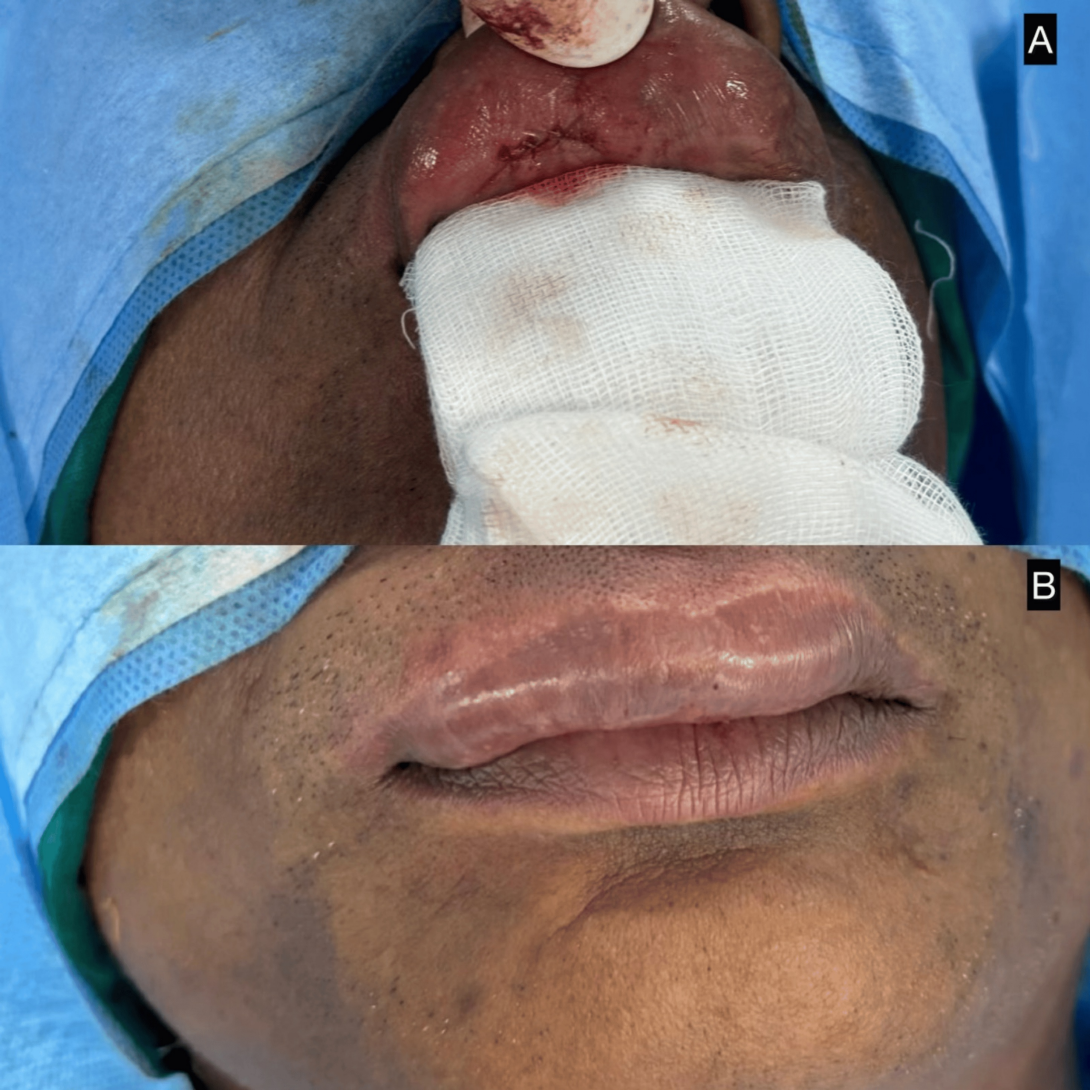
Postoperative appearance following complete excision of the upper labial sinus tract lesion A: Immediate postoperative view showing sutured surgical site; B: Immediate postoperative image after removal of gauze, showing satisfactory wound closure with good cosmetic alignment of the upper lip.

The postoperative period was uneventful, and the patient remained asymptomatic on follow-up, with no signs of recurrence (Figure 2A, 2B).

**Figure 3:**
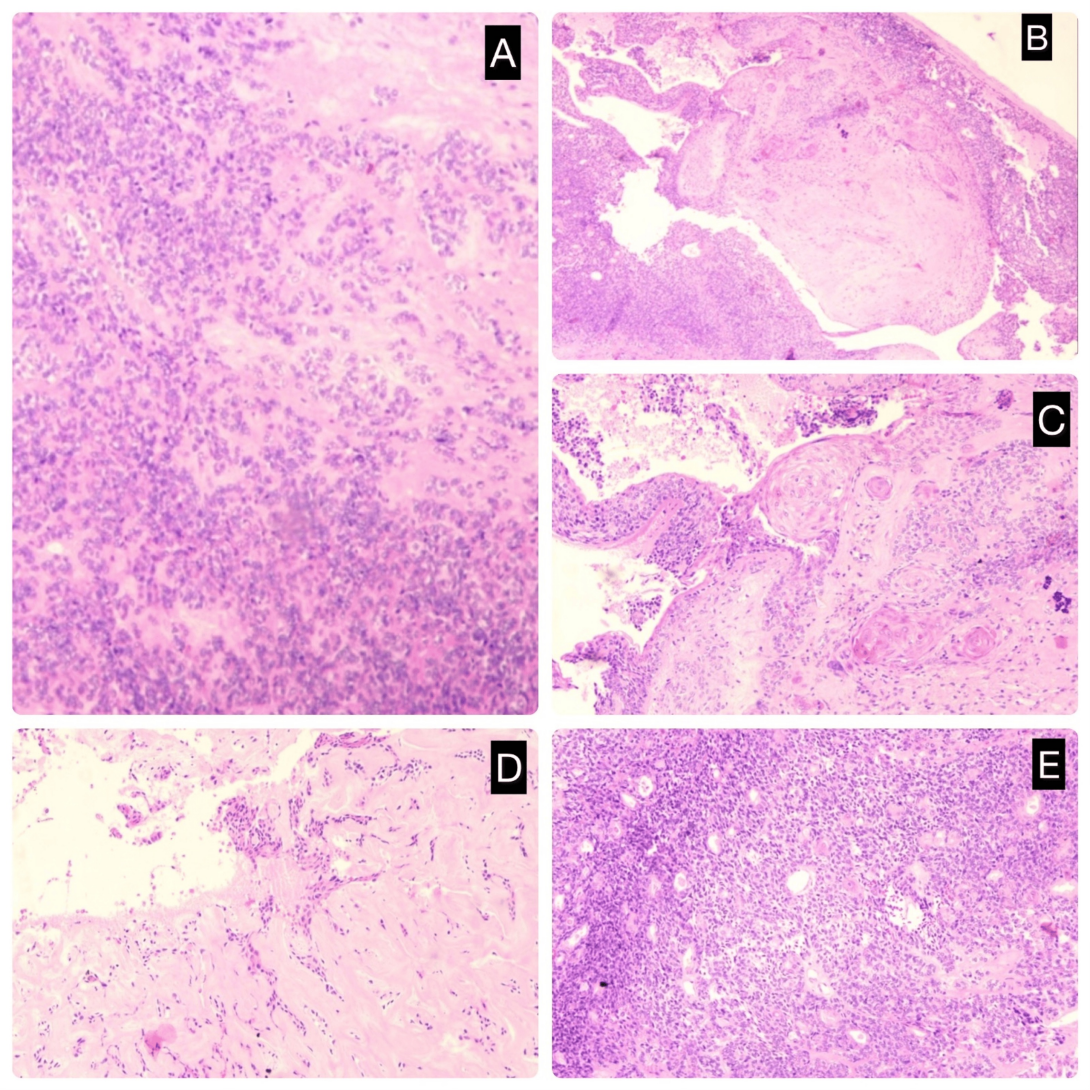
Microscopic images of a benign adnexal tumor of eccrine origin (chondroid syringoma) demonstrating key histopathological features. A: Cellular areas showing myoepithelial cells; B: Cellular areas showing myoepithelial cells and chondromyxoid areas; C: Cords of myoepithelial cells; D: Tumor cells in tubules and sheets; E: Areas of squamous metaplasia

Histopathological examination revealed a well-encapsulated lesion composed of both epithelial and myoepithelial cells arranged in sheets, cords, and tubular structures. The background demonstrated a prominent chondromyxoid matrix. Areas of squamous metaplasia and keratinous cystic changes were also identified. These findings were consistent with a diagnosis of benign mixed tumor of eccrine origin, also known as a chondroid syringoma (Figures 3A-3E).

## Discussion

Benign adnexal tumors of eccrine origin are infrequently encountered in clinical practice, particularly in the head and neck region. These tumors can mimic common entities such as epidermoid or sebaceous cysts, leading to misdiagnosis and underreporting [4].

Histologically, eccrine tumors demonstrate a combination of ductal epithelial structures and myoepithelial components embedded in a variable stromal background. Differentiation between eccrine and apocrine lineage often requires immunohistochemical studies, though routine hematoxylin and eosin staining can suffice in classical presentations [5].

The diagnosis in our case was supported by histological features, including myoepithelial cell cords and chondromyxoid stromal areas, which are characteristic of a benign mixed tumor of eccrine origin. These tumors typically exhibit low recurrence rates when completely excised surgically. Although rare, malignant transformation has been reported, particularly in long-standing or recurrent lesions [6,7]. Preoperative fine-needle aspiration cytology (FNAC) or core biopsy is not always routinely performed in small, superficial, well-circumscribed cutaneous lesions that are clinically suspected to be benign and amenable to complete excision. However, it may be considered when the lesion is deep-seated, rapidly enlarging, recurrent, located in cosmetically or functionally sensitive areas, or if malignancy is clinically suspected [1, 2]. In particular, chondroid syringomas can mimic other adnexal tumors, and FNAC may help differentiate between benign and malignant components in ambiguous cases [3]. While FNAC has limitations in fully characterizing mixed tumors due to their biphasic nature, it remains a useful preliminary diagnostic tool in selected scenarios, especially when excisional biopsy carries procedural risks.

While most reported cases involve the scalp, trunk, or limbs, upper lip presentations are extremely rare and thus merit clinical attention. The management approach remains consistent-complete excision and histological evaluation [8]. 

Differential diagnoses for chondroid syringoma include benign adnexal tumors such as dermatofibroma, epidermoid cyst, pilomatricoma, and syringoma, as well as soft tissue tumors like neurofibroma and, in rare cases, basal cell carcinoma if the lesion is pigmented or ulcerated. Clinically, these lesions may present as slow-growing, painless nodules, often indistinct without histological evaluation [5].

On investigative imaging, most of these lesions are superficial and may show nonspecific findings; however, pilomatricoma may show calcifications, and epidermoid cysts typically have a central punctum or keratin content on ultrasound. Preoperative FNAC can offer clues but may be limited in biphasic tumors like chondroid syringoma, where epithelial and mesenchymal components may not be equally sampled [7].

On histopathology, chondroid syringoma is distinguished by its biphasic architecture, with branching epithelial cords or tubules embedded in a chondromyxoid or cartilaginous stroma. This combination helps differentiate it from syringoma (which shows only small eccrine ducts in a fibrous stroma), pilomatricoma (which shows basaloid cells with ghost cells), and dermatofibroma (which lacks epithelial components entirely and shows spindle cell proliferation). Immunohistochemistry further aids diagnosis, with chondroid syringoma typically expressing cytokeratin, S-100, and epithelial membrane antigen (EMA).

The summarized clinicopathological features of chondroid syringoma are presented in Table 1 for quick reference and comparison with previously reported cases [1-8]​.

**Table 1:** Key clinicopathological features of chondroid syringoma Adapted from current case findings and supporting literature [1–8]

Parameter	Details
Tumor type	Benign mixed tumor of sweat gland (chondroid syringoma)
Typical location	Head and neck region (especially upper lip, cheek, and scalp)
Age and gender predilection	Common in middle-aged men
Clinical features	Painless, firm, slowly enlarging subcutaneous nodule
Imaging characteristics	Well-defined, hypoechoic/cystic lesion with internal debris
Histological hallmarks	Biphasic tumor: epithelial and myoepithelial components in chondromyxoid stroma
Special features	May show squamous metaplasia and keratinous cystic areas
Treatment	Complete surgical excision
Prognosis	Excellent; recurrence is rare with clear margins
Malignant transformation	Extremely rare but reported in the literature

## Conclusions

Benign mixed tumors of eccrine origin are rare but clinically significant due to their diagnostic ambiguity and potential to mimic more common lesions. Histopathological evaluation remains indispensable for definitive diagnosis. Surgical excision is curative in most cases, with an excellent prognosis and minimal recurrence risk. Clinicians should maintain a broad differential for persistent lip swellings and consider rare adnexal neoplasms in their workup.
